# SARS-CoV-2 Infection and Associated Rates of Diabetic Ketoacidosis in a New York City Emergency Department

**DOI:** 10.5811/westjem.2021.2.49634

**Published:** 2021-05-25

**Authors:** Jared Ditkowsky, Adam C. Lieber, Evan S. Leibner, Nicholas Genes

**Affiliations:** * Icahn School of Medicine at Mount Sinai, Department of Emergency Medicine, New York City; † Icahn School of Medicine at Mount Sinai, Department of Emergency Medicine, New York City; ‡ Icahn School of Medicine at Mount Sinai, Institute for Critical Care Medicine, New York City

## Abstract

**Introduction:**

In early March 2020, coronavirus 2019 (COVID-19) spread rapidly in New York City. Shortly thereafter, in response to the shelter-in-place orders and concern for infection, emergency department (ED) volumes decreased. While a connection between severe acute respiratory syndrome coronavirus 2 (SARS-CoV-2) infection and hyperglycemia/insulin deficiency is well described, its direct relation to diabetic ketoacidosis (DKA) is not. In this study we describe trends in ED volume and admitted patient diagnoses of DKA among five of our health system’s EDs, as they relate to peak SARS-CoV-2 activity in New York City.

**Methods:**

For the five EDs in our hospital system, deidentified visit data extracted for routine quality review was made available for analysis. We looked at total visits and select visit diagnoses related to DKA, across the months of March, April and May 2019, and compared those counts to the same period in 2020.

**Results:**

A total of 93,218 visits were recorded across our five EDs from March 1–May 31, 2019. During that period there were 106 diagnoses of DKA made in the EDs (0.114% of visits). Across the same period in 2020 there were 59,009 visits, and 214 diagnoses of DKA (0.363% of visits)

**Conclusion:**

Despite a decrease in ED volume of 26.9% across our system during this time period, net cases of DKA diagnoses rose drastically by 70.1% compared to the prior year.

## INTRODUCTION

The coronavirus 2019 (COVID-19) pandemic began to impact visits to our system’s New York City emergency departments (ED) in March 2020. The city’s first case was detected at our New York City ED on March 1. Case rates rapidly rose across the city; on March 12 mass gatherings in NYC were restricted, and on March 20 a “shelter-in-place” model was ordered by the governor.^1^ By April 6, COVID-19 cases peaked, and they have steadily decreased since.^2^ As public health measures went into effect, ED visits at our system’s EDs dropped significantly, and they have only recently started rising again.

COVID-19 has many pathologic manifestations. One difficult-to-manage aspect of severe COVID-19 infections is uncontrolled hyperglycemia and diabetic ketoacidosis (DKA).^3^ Patients with a history of diabetes mellitus (DM) are also at increased risk for mortality;^4^ DM was shown to be the leading risk factor among chronic medical conditions along with cerebrovascular disease for COVID-19 mortality.^5^ In several retrospective studies, uncontrolled hyperglycemia has been associated with worsening mortality,^3^ and recent consensus guidelines support the importance of glycemic control.^6^, ^7^ An exact pathophysiology for this phenomenon has not been elucidated, although several theories exist. Elevated glucose levels in pulmonary secretions are thought to suppress antiviral immune response. Furthermore, it is possible that exposure of pulmonary epithelial cells to elevated glucose concentrations increase viral replication, as it does for influenza.^4^ In this study we present retrospective findings from our own institution’s EDs that support the theory that COVID-19 infection is associated with a notable increase in concomitant DKA.

## METHODS

The hospital system’s EDs include academic and community-oriented facilities in a diverse urban environment and see over 500,000 visits a year across three boroughs in New York City. Five of these EDs are on a shared electronic health record system (Epic Systems Corporation, Verona, WI). For these EDs, deidentified visit data extracted for routine quality review was made available for analysis. The data was initially part of a quality assurance/quality improvement project and did not require institutional review board approval. We looked at total visits, and select visit diagnoses related to DKA, across the months of March, April, and May 2019 and compared those counts to the same period in 2020.

## RESULTS

**Figure 1:**
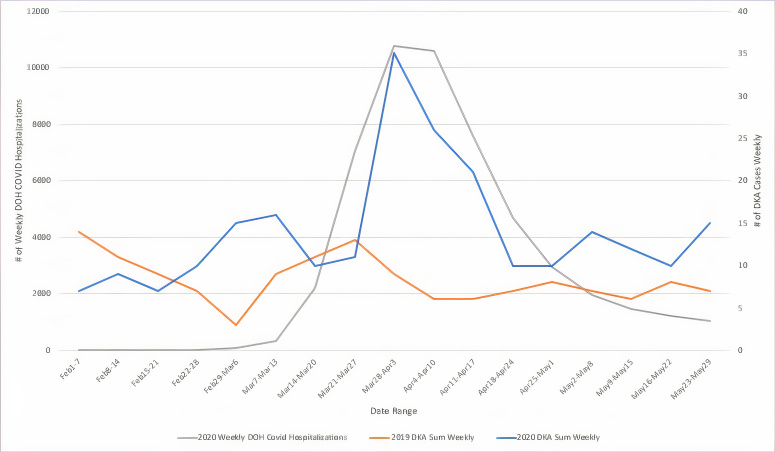
New York City weekly COVID-19 hospitalizations vs visits for diabetic ketoacidosis, 2019 vs 2020. *DOH*, Department of health; *DKA*, diabetic ketoacidosis.

**Figure 2:**
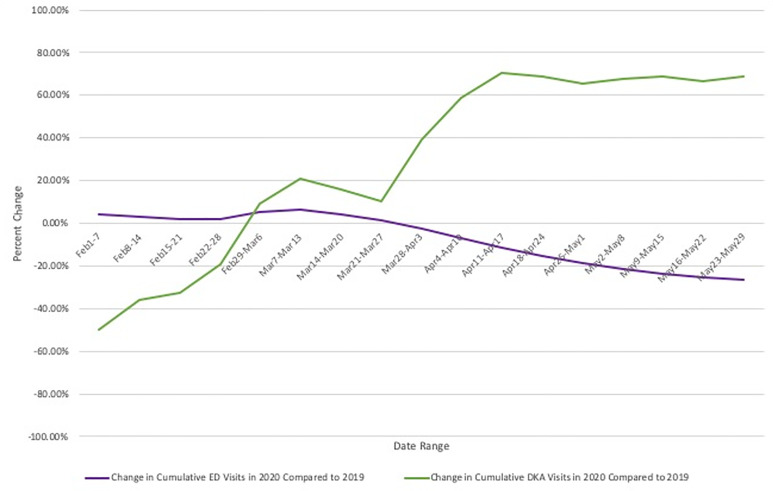
Percent change in both emergency department visits and visits for diabetic ketoacidosis between 2020 and 2019. *ED*, emergency department; *DKA*, diabetic ketoacidosis.

A total of 93,218 visits were recorded across our five EDs from March 1–May 31, 2019. During that period there were 106 diagnoses of DKA made in the EDs (0.114% of visits). Across the same period in 2020 there were 59,009 visits, and 214 diagnoses of DKA (0.363% of visits). Figure 1 compares the timeline of the NYC COVID-19 pandemic based on weekly hospitalizations as reported by the Department of Health to the observed rise in DKA visits in that same time period and compares this to DKA rates in 2019. Figure 2 displays percent change in cumulative DKA visits (what change in percent of total 2019 DKA rates was observed in 2020) compared to cumulative percent change in ED visit volume (what change in percent of total 2019 ED visits was observed in 2020). This is displayed against weekly DKA visit rates in 2019 and 2020.

## DISCUSSION

Shortly after March 1, 2020, the number of ED visits with a diagnosis of DKA began to increase across our system’s EDs, compared to the year prior. Even as daily ED visits began to drop in late March, the rate of ED DKA visits rose. This increased rate was noted throughout the period reviewed. By mid-May 2020, although ED visits were approximately one-third of those in 2019, net diagnoses of DKA approximately doubled. Similar to these findings, other authors have pointed out a correlation that suggests COVID-19 can precipitate DKA in many patients.

Several theories may explain the observed growth in DKA diagnoses during this period. Beyond physiologic mechanisms, this rise in DKA could simply represent the inability for diabetic patients to get insulin prescriptions during the public health emergency; many clinics were not able to meet regularly with patients. However, given the association between severe COVID-19 infections, hyperglycemia, and a history of DM, it is reasonable to suspect patients may present with concomitant DKA disproportionately to other disease states. Similar to other acute infections, COVID-19 infections are not only worsened by hyperglycemia, but associated with increased incidence of hyperglycemia.^8^ This may be a consequence of stress hyperglycemia from release of counter-regulatory hormones.^9^ COVID-19 is also associated with a relative insulin deficiency due to pancreatic islet cells’ ACE2 receptor, which may allow viral entry to pancreatic parenchyma leading to islet cell damage.^10^ The combination of worsened serum glucose with relative insulin deficiency may lead to an increased incidence of DKA in COVID-19 infected patients.

Given a likely shortage of intensive care unit (ICU) beds globally as a result of COVID-19, including in the United States, clinicians will need to use healthcare resources judiciously.^11^ It may be necessary to find alternate treatment strategies for treating DKA to help preserve these resources, as management often necessitates ICU level of care. Further analysis of ED visits will involve correlating DKA visits with COVID-19 test results, as well as an assessment of DKA severity and inpatient course.

## LIMITATIONS

This was a retrospective study that indicates a correlation between DKA visits and the COVID-19 pandemic; however, no causation can be established. Other factors during this period such as limited patient access to clinics may have impacted rates of DKA. The study is also geographically limited in nature, and further study will be needed to definitively state the trend is applicable to other localities. Additionally, data prior to 2019 was not available for review as additional EDs within our system were not using a shared electronic health record, leading to a temporally limited study. We were unable to assess direct rates of concomitance of COVID-19 infection and DKA.

## CONCLUSION

Despite a decrease in ED volume of 26.9% across our health system during the COVID-19 pandemic in New York City, net cases of diabetic ketoacidosis diagnoses rose drastically by 70.1% compared to the prior year. Although further study is needed, these findings may indicate a direct relationship between COVID-19 infection and risk of developing DKA.
